# Technical Assistance and Changes in Nutrition and Physical Activity Practices in the National Early Care and Education Learning Collaboratives Project, 2015–2016

**DOI:** 10.5888/pcd15.170239

**Published:** 2018-04-26

**Authors:** Alethea Chiappone, Teresa M. Smith, Paul A. Estabrooks, Cristy Geno Rasmussen, Casey Blaser, Amy L Yaroch

**Affiliations:** 1 Gretchen Swanson Center for Nutrition, Omaha, Nebraska; 2 College of Public Health, University of Nebraska Medical Center, Omaha, Nebraska; 3 Barbara Davis Center for Diabetes, University of Colorado Denver, Aurora, Colorado

## Abstract

**Purpose and Objectives:**

The National Early Care and Education Learning Collaboratives Project (ECELC) aims to improve best practices in early care and education (ECE) programs in topic areas of the Nutrition and Physical Activity Self-Assessment in Child Care (NAP SACC). Technical assistance is a component of the ECELC, yet its effect on outcomes is unclear. Beyond dose and duration of technical assistance, limited research exists on characteristics of technical assistance that contribute to outcomes. The objective of this study was to identify and describe technical assistance characteristics and explore associations with NAP SACC outcomes.

**Intervention Approach:**

We collected data from 10 collaboratives comprising 84 ECE programs in 2 states in 2015–2016. The objective of technical assistance was to support programs in improving best practices. Technical assistance was provided to programs via on-site, telephone, or email and was tailored to program needs.

**Evaluation Methods:**

We used a mixed-methods design to examine associations between technical assistance and NAP SACC outcomes. We used multiple regression analysis to assess quantitative data and qualitative comparative analysis to determine necessary and sufficient technical assistance conditions supporting NAP SACC outcomes. We also conducted a document review to describe technical assistance that referred conditions identified by the qualitative comparative analysis.

**Results:**

Regression analyses detected an inverse relationship between changes in NAP SACC scores and hours of technical assistance. No clear pattern emerged in the qualitative comparative analysis, leaving no necessary and sufficient conditions. However, the qualitative comparative analysis identified feedback as a potentially important component of technical assistance, whereas resource sharing and frequent email were characteristics that seemed to reduce the likelihood of improved outcomes. Email and resource sharing were considered primarily general information rather than tailored technical assistance.

**Implications for Public Health:**

Technical assistance may be used in programs and made adaptable to program needs. The inclusion and evaluation of technical assistance, especially tailored approaches, is warranted for environmental interventions, including ECE settings.

## Introduction

The prevalence of obesity is 9% among preschool-aged children in the United States (1). Early-life dietary habits and body weight are strongly related to risk for obesity in adulthood (2,3). Given that almost 7 million children in the United States under the age of 5 spend an average 25 hours or more per week in child care centers or Head Start, early care and education (ECE) programs are a critical setting for obesity prevention efforts (4,5). The physical and social environment of ECE programs help shape children’s physical activity and dietary behaviors; therefore, fostering effective strategies to help child care providers establish healthy environments is key (5–7).

The National Early Care and Education Learning Collaboratives Project (ECELC), funded by the Centers for Disease Control and Prevention and implemented by Nemours Children’s Health System, is using a train-the-trainer model to reduce childhood obesity by promoting healthy environments in ECE programs (8). Since its inception in 2012, the ECELC has improved ECE program practices and policies in topic areas of the Nutrition and Physical Activity Self-Assessment in Child Care (NAP SACC). However, NAP SACC outcomes at the program level vary; this variation may be attributed to dosage of components of the ECELC received by ECE programs and the ability of ECE programs to select their own goals within the intervention (9).

To address real-world issues, such as time constraints, resources, and low prioritization from providers, technical assistance was developed as a key component of the ECELC; the intent was to augment peer-to-peer training sessions in an intervention where programs select and prioritize their own goals (8,10). Technical assistance is defined as targeted or tailored support given to an individual or organization to help assist with successful development, implementation, and evaluation of a program, policy, intervention, or service through shared knowledge, resources, and expertise (11). Its role in the ECELC is to guide ECE programs as they make changes to practices and policies throughout implementation (8); the provision and quality of technical assistance may be linked to NAP SACC outcomes. Although technical assistance is not unique to the ECELC and is reported in childhood obesity prevention efforts (12–15) and other health promotion disciplines (16–18), the amount, type, and frequency of technical assistance that is necessary to support best practices for NAP SACC in ECE settings is not known.

Childhood obesity prevention research has focused on the dose and duration of technical assistance, demonstrating a relationship with outcomes. For example, one study found that the number of technical assistance sessions was associated with faster school-level improvements (13), and another study found that the number of years of technical assistance was associated with declines in overweight and obesity (19). These studies support the value of technical assistance; however, technical assistance is complex in that it can be provided in varying quantities, methods, and modes, and for different reasons (20–22). In childhood obesity prevention, research on how technical assistance contributes to successful implementation and outcomes is limited (12), and no research has examined associations between technical assistance characteristics and outcomes beyond dose and duration. This lack of research is potentially problematic if technical assistance is considered a component that contributes to the outcomes of an intervention. Because technical assistance is adaptable, technical assistance can improve program fidelity and allow local adjustments to be made according to participant characteristics, barriers, and real-world issues (12). Therefore, technical assistance should be included in analyses and discussion when it is included as a key intervention component. Exploring the characteristics of technical assistance may contribute to developing effective technical assistance models, allowing for more deliberate technical assistance provision, and allocating resources to technical assistance in health promotion programs.

## Purpose and Objectives

This descriptive study described technical assistance provided as a part of the national ECELC. Our purpose was to identify and describe characteristics of technical assistance and explore associations with NAP SACC outcomes in 5 topic areas: breastfeeding and infant feeding, child nutrition, infant and child physical activity, screen time, and outdoor play and learning (23). Our evaluation sought to determine whether the modes and methods of technical assistance in the ECELC were related to changes in NAP SACC outcomes. Findings can be used to foster continued improvement in technical assistance in the ECELC and can inform other health promotion programs that use technical assistance.

## Intervention Approach

The ECELC is implemented among cohorts (called “collaboratives”) of ECE programs in states across the United States, so that it can be evaluated for effectiveness and improvements iteratively. The intervention described in this evaluation started in October 2015 and lasted approximately 10 months. Once enrolled in the intervention, each ECE program established a leadership team that typically included 3 staff members (ie, representatives such as directors and lead teachers). Members of each leadership team participated in learning sessions, which included 5 in-person workshops, each lasting about 6 hours. Learning sessions focused on the 5 NAP SACC topic areas and included assistance in developing and implementing an action plan, a tool to support ECE programs in improving practices and policies. Between each learning session, ECE programs worked toward achieving program goals and objectives. Leadership teams facilitated training sessions to share information with ECE program staff members, executed learning session tasks, worked on implementing action plans, and received technical assistance.

This round of ECELC implementation comprised 10 collaboratives in 2 states, Missouri and Florida. Each collaborative was led by one technical assistance provider (trainer), who provided technical assistance to an average of 9 programs. Trainers provided technical assistance to ECE programs on-site or via telephone and email by using tailored approaches (eg, discussion, modeling, shared resources) according to ECE program needs. The provision of technical assistance began one month before the first learning session, continued throughout implementation, and ended one month after the last learning session.

### Participants

ECE programs that 1) served infants, toddlers, and preschoolers, 2) received technical assistance, and 3) completed the NAP SACC pre-assessment and post-assessment for all 5 topic areas were included as participants in this study. At enrollment, programs indicated whether they participated in the Child and Adult Care Food Program (CACFP) and Quality Rating and Improvement System (QRIS) and also provided information on their accreditation status, nonprofit status, and age groups served (infant, toddler, and preschool). Eighty-four programs participated (45 in Florida and 39 in Missouri); 105 programs were excluded for not meeting inclusion criteria. The primary reasons for exclusion were that programs did not serve all 3 age groups (n = 79), did not have a complete NAP SACC pre-assessment and post-assessment for all 5 topic areas (n = 88), or did not have complete technical assistance records (n = 3). Exclusion criteria were not mutually exclusive; for example, programs that did not serve infants did not complete the breastfeeding and infant feeding section of the NAP SACC assessment.

## Evaluation Methods

We used a modified explanatory sequential mixed-methods design to evaluate the technical assistance component of the ECELC, which involved collecting and analyzing quantitative data and then elucidating findings by using qualitative data (Figure) (24,25). We examined the association between hours of technical assistance and NAP SACC outcomes by using multiple regression analysis. A qualitative comparative analysis was then conducted to identify possible necessary conditions (necessary conditions would be found in all high-performing sites and also some low-performing sites) and sufficient conditions (sufficient conditions would include combinations of technical assistance found only in high-performing sites) that may support improvements in NAP SACC scores for technical assistance characteristics not included in the regression model. Preliminary analyses indicated that trainers commonly used multiple technical assistance characteristics in a single technical assistance interaction. These data were included in the qualitative comparative analysis to account for equifinality (ie, the possibility of more than one causal pathway to achieve an outcome) (26). Lastly, we conducted a document review to describe and contextualize the necessary and sufficient conditions identified by the qualitative comparative analysis. Evaluation activities were approved by the Nemours Children’s Health System Institutional Review Board.

**Figure:**
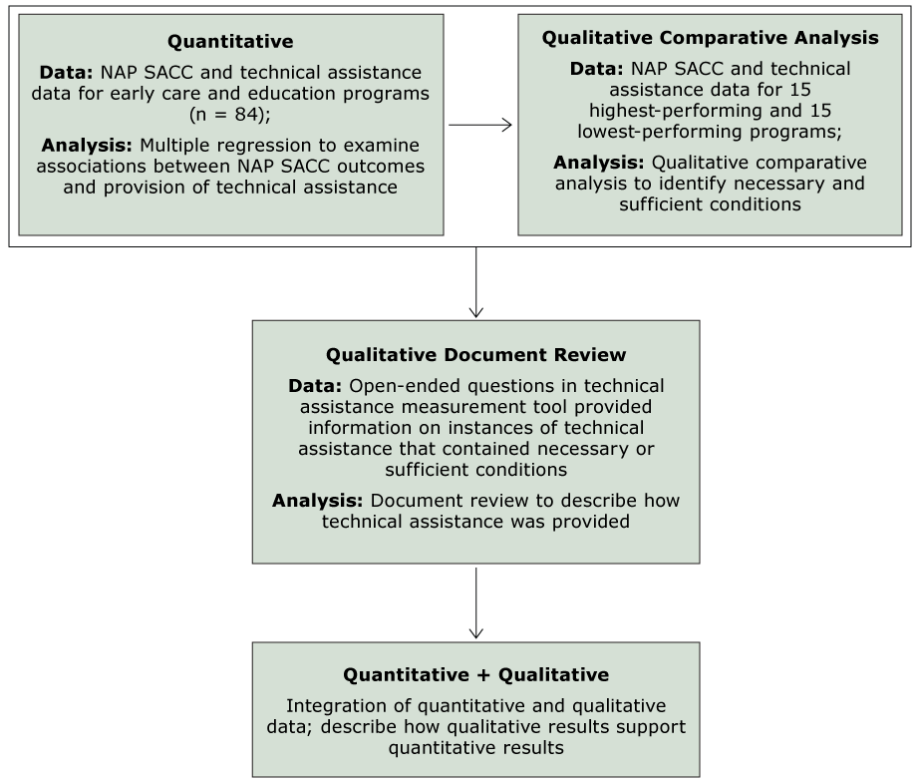
Application of a modified sequential explanatory mixed methods framework. The 15 programs with the highest ratio of possible change in NAP SACC scores were defined as high-performing programs, and 15 programs with the lowest ratio of possible change were defined as low-performing programs. Abbreviation: NAP SACC, Nutrition and Physical Activity Self-Assessment in Child Care.

### Nutrition and Physical Activity Self-Assessment in Child Care (NAP SACC)

The NAP SACC served as the main outcome measure for the ECELC. One hundred twenty-one items assessed best practices in 5 topic areas: breastfeeding and infant feeding (23 items), child nutrition (44 items), infant and child physical activity (22 items), screen time (12 items), and outdoor play and learning (20 items) (23). Items addressed policy, practices, and education and professional development (Box). Each item’s response option was on a 4-point scale ranging from noncompliance to total compliance with the best practice. For our assessment, we considered total compliance to mean that the best practice was met (score = 1) and all other responses to mean that best practice was not met (score = 0); the maximum score for a single program was 121. Leadership team members completed a paper version of the NAP SACC on-site; the pre-assessment was completed after learning session 1 and the post-assessment after learning session 4.

Box **Nutrition and Physical Activity Self-Assessment in Child Care (NAP SACC) Topic Areas and Subtopic Areas**

**Table:** 

Topic Area	Subtopic Area
Breastfeeding and infant feeding	Breastfeeding environment; breastfeeding support practices; breastfeeding education and professional development; breastfeeding policy; infant foods; feeding practices; infant feeding education and professional development; infant feeding policy
Child nutrition	Food provided; beverages provided; feeding environment; feeding practices; menus and variety; education and professional development; policy
Infant and child physical activity	Time provided; indoor play environment; daily practices; education and professional development; policy
Screen time	Availability; daily practices; education and professional development; policy
Outdoor play and learning	Outdoor play time; outdoor play environment; education and professional development; policy

### Measurement of technical assistance

We developed a novel measurement tool to capture data on the characteristics of technical assistance reported by trainers in the ECELC. Trainers recorded each instance of technical assistance by using an iPad mini (Apple Inc) and the FileMaker Go application (FileMaker, Inc). This measurement tool captured the following descriptive data: the name of the trainer who provided the technical assistance, the names of the ECE program that received the technical assistance, the date that technical assistance was provided, timing within the intervention (eg, after learning session 1), the number of minutes providing technical assistance, and the amount of travel time (eg, if technical assistance was provided on-site). The mode of delivery was recorded as on-site, telephone, email, or other. The methods of technical assistance were recorded as self-assessment, action plans or goal setting, staff training on-site, staff engagement, family engagement, observation, discussion, modeling, feedback, resource sharing, or other. Additionally, the tool asked which NAP SACC topic area(s) technical assistance pertained to and whether the technical assistance was related to that ECE program’s action plan (yes, no, don’t know). In open-ended questions, trainers had the option to describe details of the technical assistance interaction: What did you help with? What went well? What did not go well? What additional help do they need with this? Lastly, trainers recorded the proposed follow-up date and mode, if applicable.

### Statistical analysis

We used SAS version 9.4 (SAS Institute Inc) for all statistical analyses. We generated descriptive summary data (means and proportions) for site characteristics, NAP SACC scores, and number of hours of technical assistance provision. For the qualitative comparative analysis, we studied 15 high-performing programs and 15 low-performing programs. We rank-ordered programs by calculating the ratio of the change in NAP SACC score (from pre-assessment to post-assessment) to maximum possible change in NAP SACC score. Fifteen programs with the highest ratio of possible change were defined as high-performing programs, and 15 programs with the lowest ratio of possible change were defined as low-performing programs. We used *t* tests and Fisher exact tests to analyze differences between the high-performing and low-performing programs. In the full sample, we used multiple regression to explore the effect of number of technical assistance hours on change in NAP SACC score while controlling for pre-assessment NAP SACC score. Statistical significance was set at α = .05 for all comparisons and analyses.

### Qualitative comparative analysis

A qualitative comparative analysis elucidates the conditions necessary (ie, need to be present to achieve an outcome, but alone do not predict the outcome) or sufficient (ie, predict an outcome) to attain an outcome (26). We conducted a fuzzy-set qualitative comparative analysis (which assigns cases to a condition by degree of membership versus fully in or fully out) to compare characteristics of technical assistance associated with high-performing programs and low-performing programs (26). From data obtained by the technical assistance measurement tool, we assessed 2 types of conditions: mode of technical assistance and method of technical assistance. The analysis assigned membership to a condition by level of belonging, which is appropriate in analyzing data with continuous variables (ie, number of instances per mode and method) (26). We dichotomized level of technical assistance received for each type of technical assistance (ie, mode and methods). First, we estimated the mean number of instances for each type of technical assistance (eg, email, on-site). Programs receiving technical assistance below the mean level of assistance (coded as “0”) were then defined as receiving less technical assistance, and programs receiving technical assistance above the mean level of assistance (coded as “1”) were defined as receiving more technical assistance. After coding conditions, we generated a truth table to identify necessary conditions (patterns present in all high-performing programs and in some low-performing programs) and sufficient conditions (patterns present in only high-performing programs) (26,27). A truth table is a qualitative tool that displays all possible combinations of conditions that may lead to a particular outcome, listing the number of instances that are classified into each condition (26).

### Document review

We reviewed the open-ended entries submitted to gather information on context and how technical assistance was provided to the 15 high-performing and 15 low-performing programs. We conducted our analysis in Microsoft Word. Using a grounded-theory approach, one coder used frequency of coding to identify emerging themes for 3 technical assistance characteristics: email (n = 634 technical assistance instances), resource sharing (n = 499), and feedback (n = 82).

## Results

Of the 84 programs in our study, 51 (60.7%) participated in CACFP, 34 (40.5%) were accredited, and 32 (38.1%) were nonprofit (Table 1). Twelve (14.3%) participated in QRIS, and 10 (11.9%) were Head Start programs. Mean NAP SACC pre-assessment score was 48.3 (standard deviation [SD], 18.6) and mean change in score from pre-assessment to post-assessment was 16.0 (SD, 15.9). The mean number of technical assistance hours provided was 23.0 (SD, 13.9).

**Table 1:** Characteristics of a Full Sample of Programs, the High-Performing Programs^a^, and the Low-Performing Programs^b^, Participating in an Intervention of the Early Care and Education Learning Collaboratives Project, 2015–2016^c^

Characteristic	Full Sample (n = 84)	High-Performing Programs (n = 15)^a^	Low-Performing Programs (n = 15)^b^	*P* Value
Participated in Child and Adult Food Care Program, no. (%)	51 (60.7)	10 (66.7)	9 (60.0)	>.99^d^
Participated in Quality Rating and Improvement System, no. (%)	12 (14.3)	3 (20.0)	4 (26.7)	>.99^d^
Head Start, no. (%)	10 (11.9)	10 (66.7)	4 (26.7)	.33^d^
Accredited, no. (%)	34 (40.5)	5 (33.3)	8 (53.3)	.46^d^
Nonprofit, %	32 (38.1)	2 (13.3)	9 (60.0)	.02^d^
NAP SACC pre-assessment score, mean (SD)	48.3 (18.6)	50.4 (13.6)	59.6 (25.2)	.23^e^
NAP SACC post-assessment score, mean (SD)	64.3 (17.3)	85.9 (6.2)	52.8 (16.9)	<.001^e^
Change in NAP SACC score from pre-assessment to post-assessment, mean (SD)	16.0 (15.9)	35.5 (8.7)	−6.8 (15.8)	<.001^e^
Hours of technical assistance provided, mean (SD)	23.0 (13.9)	17.5 (11.4)	24.9 (15.7)	.17^e^

Abbreviations: NAP SACC, Nutrition and Physical Activity Self-Assessment in Child Care; SD, standard deviation.

a Programs with the highest ratio of possible change in NAP SACC scores from pre-assessment to post-assessment.

b Programs with the lowest ratio of possible change in NAP SACC scores from pre-assessment to post-assessment.

c Each program self-assessed 121 items in 5 NAP SACC topic areas at pre-assessment and post-assessment: breastfeeding and infant feeding (23 items), child nutrition (44 items), infant and child physical activity (22 items), screen time (12 items), and outdoor play and learning (20 items). If best practice was met, item was scored as 1; the maximum score for a single program was 121.

d Calculated by using Fisher exact test.

e Calculated by using a *t* test.

Ten of the 15 high-performing programs participated in CACFP, 10 were Head Start programs, and 2 were nonprofit. The high-performing programs received a mean of 17.5 (SD, 11.4) hours of technical assistance. Nine of the 15 low-performing programs participated in CACFP, 9 were nonprofit, and 8 were accredited; these programs received a mean of 24.9 (SD, 15.7) hours of technical assistance. Besides a significant difference in the change in NAP SACC score (high-performing programs, change = 35.5 (SD, 8.7) vs low-performing programs, change = −6.8 (SD, 15.8); *P* < .001), the only significant difference between high-performing and low-performing programs was in nonprofit status: we found significantly fewer high-performing programs with nonprofit status (*P* = .02).

Collectively, the 84 programs received 3,100 instances of technical assistance during the study period. Hours of technical assistance significantly predicted changes in NAP SACC scores, after we controlled for pre-assessment score (β = −0.22, *P* = .049). In this model, each additional hour of technical assistance, regardless of type, was associated with a decrease of 0.22 best practices. The truth table showed several patterns that seemed to facilitate high performance and other patterns that seemed to inhibit high performance (Table 2). Specifically, technical assistance provided through feedback was identified in 7 high-performing programs but in only one low-performing program. The truth table also indicated that email and resource sharing might inhibit high performance.

**Table 2:** Truth Table^a^: Cross-Program Comparison of Characteristics of Technical Assistance Provision That May Affect NAP SACC Outcomes^b^

Characteristic	By Site															Total
**High-Performing Programs^c^**																
**Site identifier**	**1**	**2**	**3**	**4**	**5**	**6**	**7**	**8**	**9**	**10**	**11**	**12**	**13**	**14**	**15**	—
**Mode of technical assistance**																
On-site	0	1	1	1	1	1	0	0	1	0	1	0	0	0	0	7
Telephone	0	1	0	0	1	1	1	1	0	0	0	1	0	1	1	8
Email	1	0	0	0	0	0	0	1	0	1	0	1	1	0	1	6
**Methods of technical assistance**																
Self-assessment	0	0	1	0	0	1	1	0	1	0	1	0	0	1	0	6
Action plans or goal setting	0	1	1	0	1	1	1	0	1	0	1	0	1	1	0	9
Staff training on-site	0	0	0	1	0	1	1	0	0	0	1	1	0	1	0	6
Staff engagement	0	1	0	1	1	0	1	0	0	0	1	0	0	0	0	5
Family engagement	0	1	0	0	1	0	1	0	0	0	1	0	0	0	0	4
Observation	0	1	1	0	1	1	0	0	1	1	0	0	0	0	0	6
Discussion	0	1	1	0	1	1	0	1	1	0	0	0	0	1	1	8
Modeling	0	1	0	0	1	1	0	0	0	1	0	0	0	0	0	4
Feedback	0	1	1	0	1	1	1	0	1	0	1	0	0	0	0	7
Resource sharing	1	0	0	0	0	0	0	1	0	1	0	1	1	0	1	6
**Low-Performing Programs^d^**																
**Site identifier**	**70**	**71**	**72**	**73**	**74**	**75**	**76**	**77**	**78**	**79**	**80**	**81**	**82**	**83**	**84**	**—**
**Mode of technical assistance**																
On-site	1	0	0	1	0	1	1	0	0	0	0	0	1	0	0	5
Telephone	1	0	1	0	0	0	0	1	1	0	0	0	0	1	0	5
Email	1	1	1	1	1	0	0	1	1	1	0	1	0	0	1	10
**Methods of technical assistance**																
Self-assessment	1	0	1	0	0	1	1	1	1	0	0	0	0	0	0	6
Action plans or goal setting	1	0	0	0	0	1	0	0	0	1	0	1	1	1	1	7
Staff training on-site	0	0	1	1	1	0	0	0	1	1	1	0	0	1	0	7
Staff engagement	1	0	1	1	0	0	1	1	1	0	0	0	0	1	0	7
Family engagement	1	0	1	0	0	0	1	1	0	0	0	0	0	1	0	5
Observation	0	1	0	0	0	1	1	0	0	0	0	0	0	0	0	3
Discussion	0	0	0	0	0	1	1	1	0	0	0	0	1	1	0	5
Modeling	0	1	1	0	0	1	0	0	0	0	0	0	0	0	0	3
Feedback	0	0	0	0	0	1	0	0	0	0	0	0	0	0	0	1
Resource sharing	0	1	1	1	1	0	1	1	1	1	1	1	0	0	1	11

Abbreviation: NAP SACC Nutrition and Physical Activity Self-Assessment in Child Care.

a A truth table is a qualitative tool that displays all possible combinations of conditions that may lead to a particular outcome, listing the number of instances that fall into each condition (26).

b Programs receiving technical assistance below the mean level of assistance (coded as “0”) were defined as receiving less technical assistance, and programs receiving technical assistance above the mean level of assistance (coded as “1”) were defined as received more technical assistance.

c Each program self-assessed 121 items in 5 NAP SACC topic areas at pre-assessment and post-assessment. Programs with the highest ratio of possible change in NAP SACC scores from pre-assessment to post-assessment were categorized as high-performing. We rank-ordered programs by calculating the ratio of the change in NAP SACC score (from pre-assessment to post-assessment) to maximum possible change in NAP SACC score. For example, the first-ranked program had a pre-assessment score of 40 and a post-assessment score of 88, with a change score of 48. The program’s possible change in the positive direction was 81 (121 − 40), for a ratio of 0.59 (48/81).

d Each program self-assessed 121 items in 5 NAP SACC topic areas at pre-assessment and post-assessment. Programs with the lowest ratio of possible change in NAP SACC scores from pre-assessment to post-assessment were categorized as low-performing. For example, the last-ranked program had a pre-assessment score of 105 and a post-assessment score of 59, with a change score of −46. Because this program’s change was in the negative direction, their possible change in the negative direction was 105, for a ratio of −0.44 (−46/105).

Technical assistance provided through email was found in 6 high-performing programs and 10 low-performing programs. Similarly, technical assistance provided through resource sharing was identified in 6 high-performing programs and 11 low-performing programs. However, the qualitative comparative analysis did not reveal the necessary or sufficient conditions to ensure high performance (Table 2).

### Technical assistance through feedback

**Collaboration.** The mode of technical assistance using feedback methods tended to be in-person or via telephone. Trainers described collaborating with program staff, which commonly, but not always, included members of the leadership team. For example, some communication was with teachers or kitchen aides. Collaboration generally aimed to facilitate programs in implementing ECLEC but also focused on the unique characteristics of ECE programs. For example, one trainer documented, “We brainstorm ways to sustain the changes made thus far. We also discussed ways to continue to engage families to continue healthy choices at home.” An example of considering unique characteristics of ECE programs is exemplified by a statement by one trainer, “Met with director and leadership member in the infant room and began rearranging to help allow for more active floor time for the infants.”

**Intervention components.** Technical assistance provided through feedback frequently focused on action plans, learning session information, and NAP SACC topic areas. Trainers provided feedback on executing action plans and reaching action period goals. One trainer documented, “Reviewed action plan goals with teacher, started the discussion on what toys/materials would be helpful to meet goal and activities to use with them.” Throughout these interactions, trainers discussed NAP SACC topic areas. This was exemplified by “We discussed doing staff tastes of new foods and also have the staff write menu suggestions on chart paper in the break room.”

### Technical assistance through sharing resources

**Type.** Commonly shared resources included newsletters, toolkits, tip sheets, websites, policy information, and recipes. Newsletters included those from ECELC and newsletters from outside of ECELC. Toolkits and tip sheets typically provided information on physical activity and nutrition best practices in ECE programs. One trainer documented, “I emailed a ‘choose MyPlate tip sheet’ supporting everyone active.” Sharing websites typically included increasing awareness of the website, increasing awareness of resources available on the website, and showing programs how to navigate websites. For example, “Walked them step by step through the LMCC [Let’s Move! Child Care] website.” Policy information included sample policies, how to create policies, and policy updates. For example, “I sent out an email with the latest Childhood Obesity Policy Update from the RWJF [Robert Wood Johnson Foundation].” Lastly, trainers shared resources with recipe indexes and individual recipes.

**Purpose.** Trainers explained why they chose to send a certain resource to a program. Two common reasons were that the trainer thought the resource was notable and aligned with intervention goals. One trainer noted the following about a resource for an outdoor play environment: “Programs have been working on these goals and this will help support them.” Other cited reasons included the following: resource supports best practices, program(s) were interested in a topic, and seasonality (eg, healthy treats during the holidays). Overall, trainers tended to send resources because they were applicable; documented requests for information made by programs were less frequent.

### Technical assistance by email

**Resources.** One of the main methods for delivering technical assistance was via email and often included sharing informational resources. Common resources trainers described sharing included newsletters, toolkits, websites, policy information, recipes, and tip sheets. Informational resource sharing was exemplified by these comments: “Resources/websites/articles that identify how foods help your body. *Washington Post* [articles about] rainbow foods (2 articles on this topic), and information from A Healthier Michigan website that discusses maximizing your health with good foods,” and “Identified healthy eating, physical activity, and gardening websites that include tips, information, tools, ideas, and sample policies related to those topics.”

**Opportunities.** Sharing funding and training opportunities was less common than provision of informational resources yet still an emergent theme. Trainers provided information about an array of training opportunities within and outside of the ECELC. Webinars were the most commonly cited form of training promoted. Reasons for sending information on training opportunities were typically that training supported best practices. Trainers also documented sharing resources for additional funding that would further support implementation and best practices outside of the NAP SACC categories. For example, one trainer documented, “Provided information on a grant that providers can apply for to further support breastfeeding moms.”

**Support.** Providing direct ECELC implementation support via email was documented less frequently. Providing support through feedback, suggestions, review, and reminders was documented. Feedback tended to focus on action plans and program goals. One trainer documented, “Reviewed action plan submitted by participant and provided feedback on action steps.” Suggestions were similar to feedback but differed in that they were more focused on active implementation. For example, “Gave some ideas and suggestions for LS5 [learning session 5] storyboards.” Trainers also sent emails that reviewed pertinent information from learning sessions. For example, “Reviewed what they should be working on for this action period.” Lastly, trainers offered reminders about implementation tasks and upcoming events. One trainer documented, “Sent out a reminder to all programs about items that need to come back with them to LS2 [learning session 2].”

## Implications for Public Health

The purpose of this study was to identify and describe characteristics of technical assistance provided in the ECELC and explore associations between characteristics and NAP SACC outcomes. The amount of technical assistance received was negatively related to changes in best practices being met, which is contradictory to evidence suggesting that technical assistance dose is positively associated with intervention outcomes (13,19). However, Madsen and colleagues reported that dose was significant in reducing overweight and obesity when, and only when, technical assistance was provided by national experts (19). These findings suggest the quality of technical assistance, and not necessarily dose, may determine the effect of technical assistance on intervention outcomes, warranting further exploration of the quality of technical assistance provided to the ECE programs in our sample. An alternative hypothesis is that ECE programs that were struggling to implement best practices requested and received a higher volume of support and that the effect of high-volume training on NAP SACC outcomes is delayed. In either case, a better understanding of the content, focus, and original reason for technical assistance contacts is needed to improve NAP SACC outcomes and provide a valuable direction for future research.

In our qualitative comparative sample, high-performing programs and low-performing programs differed in the proportion of nonprofit ECE programs. More low-performing programs than high-performing programs were nonprofit. Nonprofit ECE programs tend to be located in low-income areas (28), which may suggest additional implementation barriers (eg, resources). Although a single study is insufficient to confirm the relationship between profit status and outcomes, training approaches may need to be tailored toward nonprofit ECE programs with a goal of stronger fidelity to best practices. Our qualitative comparative analysis did not reveal any necessary or sufficient conditions. However, results of studies may vary according to how qualitative comparative analysis methods are applied (eg, identifying cases) (26), suggesting a need for researchers to consider characteristics of the data (eg, sample size) in the application of appropriate methods. In our study, we considered reducing the number of conditions to prevent over-specification (ie, overfitting of truth tables) (29), but because technical assistance is complex, we determined it appropriate to not limit the number of conditions.

Our findings showed that feedback was a potentially important component of technical assistance. As described in the open-ended entries submitted by trainers, technical assistance provided though feedback was targeted at a program’s unique characteristics, addressed ECELC intervention components (eg, an action plan), and included collaboration with ECE program staff members. This finding supports current technical assistance models, which suggest that successful technical assistance includes both content-driven and relationship-based elements (20). Important service-delivery features include awareness of context, flexibility, and engagement (20). In our study, technical assistance provided through feedback emphasized *fit* and *what works*. Tailoring adjustments to reduce implementation barriers is an advantage of technical assistance (12) and may also assist in forming strong partnerships, which was viewed as promoting successful technical assistance (21).

Our study showed that email and shared resources, which were commonly web-based, were identified as conditions that may impede outcomes. Technical assistance provided by email and shared resources was commonly described by trainers as general information sharing (eg, newsletters, fact sheets) and funding announcements. Considering cited implementation constraints, such as time and resources (10), and that surveyed ECE providers reported the internet and short 1-day conferences as preferences for receiving information (30), one might expect that web-based technical assistance may alleviate barriers and improve implementation. However, our document review elucidated that technical assistance provided by email and shared resources commonly included generalized information targeting overall ECELC goals, not individual program goals. To improve resource sharing and email correspondence, it would be valuable to consider providing information tailored to ECE needs and provide feedback and “how-to” support for implementation of new concepts or activities at the ECE program (31).

Our evaluation study has some limitations. First, the NAP SACC was not originally intended as an outcome measure. Because the NAP SACC pre-assessments and post-assessments were administered after learning session 1 and learning session 4, respectively, outcomes are not a true reflection of pre-intervention and post-intervention. However, a previous study demonstrated that 89% of NAP SACC items showed at least moderate agreement for test–retest reliability, 100% showed at least moderate agreement for inter-rater reliability, and 52% showed at least moderate agreement for validity when tested against the Environment and Policy Assessment and Observation instrument (κ ≥ 0.20) (32). In addition, fuzzy-set qualitative comparative analysis assigns cases to a condition by degree of membership (26), which in our evaluation study was below the mean or above the mean. A drawback is that the mean is considered an arbitrary cut-off point. The alternative, a crisp-set qualitative comparative analysis, which establishes membership to a condition as fully in or fully out (26), would not have been appropriate because our variables were continuous.

Despite these limitations, this study has several strengths. The mixed-methods approach allowed us to further explain and understand quantitative findings (eg, number of instances of technical assistance) and describe the variability in quality of technical assistance. Further, these findings add to the current literature on technical assistance, emphasizing that the fit within a program’s context is important to consider for successful implementation. Future programming should consider how content-driven technical assistance could be more targeted (ie, directed at program needs) and delivered more effectively (eg, personalized correspondence vs group email). Furthermore, providing targeted content-driven technical assistance within the context of building and/or maintaining relationships may strengthen the impact of technical assistance, but this idea needs to be tested. One hypothesis derived from this evaluation study is that overly generic technical assistance — technical assistance that is not tailored to a program’s unique needs and goals — may dilute the effect of technical assistance (33), though the field of public health as a whole needs to better define and characterize technical assistance. Other factors may affect outcomes in ECE settings. For example, a program’s readiness to change may affect the provision of technical assistance, so future research should compare how technical assistance can be effectively used among programs with low levels of readiness and high levels of readiness. Programs motivated to change may be able to do so with minimal technical assistance, whereas one that is less motived or has greater perceived barriers to change may need more technical assistance to move in a positive direction. Additional research may reveal how interventions can develop effective technical assistance models, provide more deliberate technical assistance, and allocate resources efficiently.

This descriptive study is one of the first to examine the effect of characteristics of technical assistance on outcomes for variables other than dose and duration. This evaluation was novel in that it employed both probabilistic and qualitative methods to address limitations of each method. It is well accepted that technical assistance is useful in improving the quality of program implementation aimed at improving health behaviors and preventing childhood obesity (9,11,18,20,21). Our evaluation study suggests that on-site or in-person tailored technical assistance, while more resource intensive, may more effectively improve program implementation quality.
